# A National Study of the Rate of Benign Pathology After Partial Nephrectomy for T1 Renal Cell Carcinoma: Should We Be Satisfied?

**DOI:** 10.3390/cancers16203518

**Published:** 2024-10-17

**Authors:** Luna van den Brink, Tess Debelle, Lieke Gietelink, Niels Graafland, Annebeth Ruiter, Axel Bex, Harrie P. Beerlage, R. Jeroen A. van Moorselaar, Brunolf Lagerveld, Patricia Zondervan

**Affiliations:** 1 Department of Urology, Amsterdam UMC, University of Amsterdam, 1105 AZ Amsterdam, The Netherlands; 2 Department of Urology, Amsterdam UMC, Vrije Universiteit Amsterdam, 1081 HV Amsterdam, The Netherlands; 3 Cancer Center Amsterdam, Imaging and Biomarkers, 1081 HV Amsterdam, The Netherlands; 4 Department of Urology, Spaarne Gasthuis, 2035 RC Haarlem, The Netherlands; 5 Department of Urology, Netherlands Cancer Institute (NKI), 1066 CX Amsterdam, The Netherlands; 6 Department of Urology, OLVG, 1091 AC Amsterdam, The Netherlands; 7 Department of Urology, Royal Free Hospital, London NW3 2QG, UK

**Keywords:** renal cell carcinoma, partial nephrectomy, pathology, T1a-b, treatment

## Abstract

**Objectives**: To determine the rate of benign pathology in cT1 tumors following partial nephrectomy in the Netherlands, thereby evaluating the rate of overtreatment. **Methods:** Data were collected from a nationwide database containing histopathology of resected renal tissue from 2014 to 2022. Patients who underwent partial nephrectomy for suspected RCC staged T1a-b were extracted for analysis. Data are shown in percentages, and multivariable logistic regression was performed to determine predictive factors for benign pathology. **Results**: 3409 cases were analyzed, of which 403 (12%) were benign and 3006 (88%) malignant. Subtype analysis showed 2126 (62%) cases of clear-cell RCC, followed by 604 (18%) of papillary RCC and 344 (10%) oncocytomas. Mean age was 63 years among patients with malignant pathology versus 65 years for patients with benign lesions (*p* < 0.001). Mean tumor size was 3.2 cm for malignant pathology and 2.9 cm for benign (*p* < 0.001). The rates of benign and malignant pathology did not change between 2014 and 2022 (*p* = 0.377). Multivariable regression showed age ≥ 65 years (65–79 years [OR 1.881, *p* = 0.002], ≥ 80 years [OR 3.642, *p* < 0.001]) and tumor size (OR 0.793, *p* < 0.001) as predictors for benign pathology. The main limitation of this study is that we do not know the biopsy rate of our cohort. **Conclusion:** This study reports a low rate of 12% benign pathology after partial nephrectomy in the Netherlands. It remains debatable whether these rates are acceptable, or if renal tumor biopsies should be utilized more frequently to reduce overtreatment.

## Simple Summary

In this national study, we found a low rate of 12% benign pathology after partial nephrectomy in the Netherlands. This raises the questions of whether we should aim to further reduce the rate of benign pathology, what rate is considered acceptable, and whether the more frequent use of renal tumor biopsies can contribute to this. We recommend that patients with clinical suspicion for renal cancer are discussed in multidisciplinary tumor board meetings to determine whether a biopsy could be of added value to avoid overtreatment.

## 1. Introduction

Renal cell carcinoma (RCC) accounts for approximately 3% of all malignancies worldwide, with an estimated 431,288 new cases in 2020, and has the highest prevalence in Western countries [1,2]. The vast increase in the use of cross-sectional imaging has resulted in an approximate 2% annual increase in the incidence of RCC per year, yet mortality rates have remained relatively unchanged [1,3,4].

More than half of all renal masses are found incidentally through medical imaging, and are otherwise known as incidentalomas [3,5]. The increased rate of incidentalomas has led to a stage migration favoring T1 tumors, whereby half of all renal masses are classified as T1a or T1b at the time of diagnosis [6]. Tumors with these classifications tend to have slower growth rates and better overall survival rates than more advanced tumors, and studies have shown that smaller renal masses have a higher likelihood of benign pathology [6,7].

Renal masses are diagnosed and characterized with the use of imaging techniques; however, the preoperative evaluation of their histopathology can only be determined with the use of a renal tumor biopsy (RTB) [3,4,6,8,9]. Although RTBs are helpful in diagnosing the histopathology of the renal mass and therefore could contribute when considering treatment options, preoperative RTBs are not standard practice. The European Association of Urology (EAU) recommends RTB for patients undergoing active surveillance (AS) and prior to ablative treatment [4]. For patients undergoing (partial) nephrectomy, RTBs are often not deemed necessary [3,4,10,11]. 

Recent evidence suggests that 8–30% of surgically removed renal masses have benign pathology, dependent on tumor size and country where the study was performed [6,7,12,13,14,15]. A direct correlation between smaller tumor size and an increased chance of benign pathology, lower grade malignancy, lower pathological stage, slower growth rate and lower risk of metastasis was found [7]. The aim of this study is to determine the rate of benign pathology in patients who underwent partial nephrectomy (PN) for T1 renal masses throughout the Netherlands and evaluate the rate of overtreatment. The secondary aim is to identify predictive factors for benign pathology in patients with cT1 renal masses.

## 2. Materials and Methods

### 2.1. Study Population

In this national retrospective cohort study, all partial nephrectomies performed on renal masses staged cT1a-b between 1 June 2014 and 31 December 2022 throughout the Netherlands were analyzed. Patient data were extracted from *PALGA*, a national archive containing histopathological samples. The database used in this study contains histopathology of resections of renal tissue in the Netherlands. Data containing histopathological information were provided via a predefined protocol utilized by pathologists from institutions across the Netherlands. This protocol has been used regularly by pathologists since 2014, thus forming the rationale to include data starting in 2014. Patients who underwent PN for suspected RCC that were staged cT1a or cT1b were extracted from the database. Patients aged 18 years or older at the date of surgery were included. Metastases from another primary malignancy or tissue that did not comprise renal cells were excluded. Data from the first PN per patient were used, thus all secondary PNs or re-excisions were excluded. If multiple tumors were removed per PN, only data of the tumor with the greatest maximal diameter were used. 

Tumors were categorized as benign and malignant based on histopathological reports. The following variables per patient were collected: age, T stage and tumor size. Patients were divided into age groups of <50 years, 50–64 years, 65–79 years and ≥80 years old. All cases were classified into histopathological subtypes.

### 2.2. Outcomes

The overall prevalence of pathologic findings was reported as a proportion. The other outcomes were the predictive factors for benign pathology, such as age, gender and tumor size. In addition, the trend of malignant and benign pathologic findings was followed over time using the date of surgery.

### 2.3. Statistical Analysis 

All statistical analyses were conducted using SPSS software (SPSS 28.0.1.1, Armonk, NY, USA). All results were expressed as percentages (%) or means with standard deviations. T-tests were used to compare the benign and malignant groups and Pearson’s Chi squared test was used for categorical data.

Univariate and multivariable logistic regression was used to determine predictive factors for benign pathology. All statistical tests used were two-sided and considered statistically significant when *p* < 0.05. 

## 3. Results

**Figure 1:**
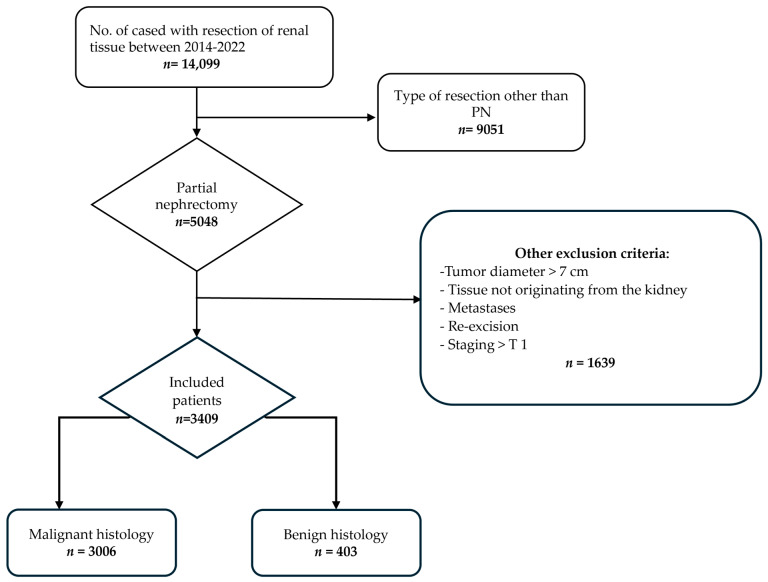
Selection of the study population.

Data were extracted from a database containing all cyto- and histopathology of renal tissue in the Netherlands between 1 June 2014 and 31 December 2022 (n = 14,099). After exclusions (n = 10,687), n = 3409 cases remained for analysis. An overview of the selection process is shown in Figure 1.

Of the 3409 cases, 3006 (88%) had malignant pathology and 403 (12%) were benign tumors. Overall, 1199 (35%) patients were female and 2213 (65%) were male. There was no difference in distribution of gender between malignant and benign lesions (*p* = 0.359). Mean age was higher in the group with benign pathology (65 years versus 63 years, *p* < 0.001). Among patients aged <50 years, 92% of cases were malignant versus 8.0% benign. In the age group of 50–64 years, 90% of cases were malignant and 10% benign. Among patients aged 65–79 years, 87% had malignant pathology compared to 13% benign, and in patients ≥ 80 years, 79% of cases were malignant compared to 21% benign. Within the entire cohort, 2674 (78%) cases were staged cT1a and 735 (22%) cT1b. A significantly higher percentage of cT1b tumors had malignant pathology (87% cT1a vs. 91% cT1b, *p* = 0.003). The mean tumor size (cm) was 3.2 cm within the entire cohort, and, when subcategorized into benign and malignant categories, the cases with malignant pathology (3.2 cm) had a larger mean tumor size than benign pathology (2.9 cm) (*p* = 0.003). Baseline characteristics are shown in Table 1.

The tumor types with the highest prevalence were clear cell RCC (62%), followed by papillary RCC (18%) and oncocytoma (10%). Histopathological subtypes are shown in Supplementary Table S1.

There was no difference in the rates of malignant and benign pathologic findings over time (*p* = 0.377), as shown in Supplementary Figure S1.

Multivariable regression showed that age groups 65–79 and ≥80 years (OR 1.881, CI 1.272, 2.781, *p* = 0.002 and OR 3.642, CI 2.030, 6.534, *p* < 0.001, respectively) and smaller tumor size (OR 0.793, CI 0.724, 0.868, *p* < 0.001) are predictive factors for benign histology. Results are shown in Table 2.

## 4. Discussion

This study gives an insight into the rate of benign pathology after PN in the Netherlands. The rate of benign pathology was 12% in our study (n = 3409), which is relatively low compared to previous studies [3,12,16,17,18], as shown in Table 3. This rate remained relatively stable between 2014 and 2022. 

Several studies have documented rates of benign pathology after PN. A retrospective, national study in the US with a cohort of 18,060 patients who underwent PN recorded an overall prevalence of benign pathology of 30.9% and found that this rate remained stable between 2007 and 2014 [17]. A meta-analysis including 47 studies reported a benign pathology rate of 19% after PN [13]. The higher rate of benign pathology in other studies [3,12,16,17,18] could be explained by a less frequent application of preoperative RTBs for renal masses. For instance, in the study by Kim et al., RTB was applied in only 7.6% of patients [17]. Contrarily, a Dutch retrospective cohort study (n = 714) including patients who underwent RTB, either before or concurrently with ablation, demonstrated a potential rate of overtreatment of 32.5% in patients who received RTB at the time of the procedure. This rate was significantly higher than the 17.7% observed in patients who underwent biopsy prior to ablation (*p* < 0.001). In addition, this study reported adverse events related to RTB in only 0.4% of patients [19]. Furthermore, biopsies present a safer alternative compared to surgery, with a lower risk of complications in comparison to PN [3,8,20]. Moreover, a case series by Macklin et al. reported tumor seeding in the tract of the RTB in only seven patients who underwent (partial) nephrectomy (n = 585) at a tertiary center. No apparent risk factors for tract seeding were identified apart from papillary subtype, and one of these seven patients developed a local recurrence. Despite this series, the clinical significance of tract seeding is still debatable [21]. A recommendation would be to discuss patient cases at multidisciplinary tumor board meetings, which have the potential to foster a more deliberate application of RTBs, thereby lowering the risk of overtreatment. 

Nonetheless, our data indicate that the prevalence of benign pathology remained constant from 2014 to 2022. A retrospective, national study by Yildirim et al. demonstrated a shift from radical nephrectomy (RN) to PN as treatment for cT1b tumors in the Netherlands between 2014 and 2020 [22]. The increasing trend towards PN for the surgical resection of renal masses, combined with the rising incidence of cT1 RCC reported by the Netherlands Cancer Registry (NCR), could explain the stable rate of benign pathology in the Netherlands [23]. Furthermore, it is questionable whether urologists should aim to further reduce the rate of benign pathology by increasing the use of RTB or if we should accept the current circumstances. 

In our study, most benign lesions were oncocytomas (10%), followed by angiomyolipomas (AMLs; 1.3%). AMLs can often be distinguished from RCCs by their characteristic appearance due to relatively high fat density on computed tomography [24]. Nonetheless, small lesions (<3 cm) or lipid poor AMLs can still be difficult to distinguish from RCC. Other studies also report a higher prevalence of oncocytomas compared to AML among benign pathology after PN [18,25,26]. Despite this, Jeon et al. reported 9.3% of AMLs and 2.9% of oncocytomas after PN in a cohort of 367 patients with cT1a renal masses [12]. This study had a high proportion of female patients (39.8%) with a benign tumor, thus this finding could also be explained by the association between AML and the female gender [27]. The retrospective study by Vijay et al. found that patients with benign pathology were more likely to be female, have a tumor size ≤ 2 cm and are more likely to have multifocal disease [28]. Evidence for the association between gender and benign pathology remains conflicting and was not found in our study [13,16,29].

Interestingly, one of the findings in our study is the association between age > 65 years and benign pathology. This outcome is confirmed by the study by Kim et al., where 35.9% of patients aged > 65 years had benign pathology compared to 29.6% of patients aged < 65 years. Multiple logistic regression showed higher age was associated with benign findings after PN (OR 0.99, CI 0.99–0.99, *p* < 0.001). These results have implications for clinical practice, as studies show that cancer specific survival does not differ between PN and active surveillance in elderly patients with small renal masses [30]. In addition, small renal masses tend to have slow growth and progression patterns, making surgical intervention in elderly and frail patients less urgent [30]. Based on these findings, we recommend that urologists should discuss active surveillance in conjunction with RTB as a treatment option for elderly patients and those who are poor surgical candidates.

This study provides a national representation of patients who underwent PN using real-world data, thereby providing a realistic overview of the benign pathology rate compared to single-center studies or randomized studies. This study also provides more recent data compared to the existing literature. Moreover, an important element of real-world data is that it reflects practice variation. For example, in the Netherlands, high volume hospitals perform PN more often for cT1b tumors compared to lower volume hospitals, and there appears to be an increasing trend toward active surveillance in patients with cT1a tumors [22]. Ultimately, these fluctuations in the management of T1 RCC over time can influence the benign pathology rate after PN. 

Our study is not free of limitations. For instance, during the validation process involving crosschecks on the raw data obtained from *PALGA*, discrepancies were found regarding the Fuhrman grade. In consequence, this information was not reliable and could not be used for analysis. In addition, we were not able to compare the benign pathology rate with the number of RTBs performed for cT1 tumors in the Netherlands using this database, so we do not know if this has increased in the past years. Lastly, we used pathological data that were registered in *PALGA* via a specific protocol. Despite the frequent application of the protocol throughout the Netherlands, there remains some selection bias. 

Furthermore, in order to reduce overtreatment and improve renal cancer care, adequate registration is essential. For example, the British Association of Urological Surgeons (BAUS) audit also investigated PNs performed in the UK in 2012 and found a benign pathology rate of 18%. In the Netherlands, the recently initiated PRO-RCC cohort currently registers all new renal cancer patients; this way, the benign pathology rate can be monitored continuously and more in-depth analyses of potential factors of influence can be performed [31].

## 5. Conclusions and Future Work

This study provides an insight into the rate of benign pathologic findings following PN in the Netherlands, which is low compared to previous studies and has remained stable between 2014 and 2022. Furthermore, these findings raise the questions of whether we should aspire to reduce the rate of benign pathology, and what rate is considered acceptable. We recommend that patients are discussed in multidisciplinary tumor board meetings to determine whether RTBs could be of added value to avoid overtreatment. Accurate registration, on both a national and international scale, of clinical practice in renal cancer is of importance to gain further insights on potential overtreatment.

## Figures and Tables

**Table 1:** Baseline characteristics of the study population.

	Total (n = 3409)	Malignant (n = 3006)	Benign (n = 403)
**Gender**			
Female (%)	1199 (35)	1049 (88)	150 (12)
Male (%)	2210 (65)	1957 (88)	253 (12)
**Age** (mean, sdev)	63 (11)	63 (11)	65 (10)
**Age category** (%)			
Age < 50 years	399 (12)	367 (92)	32 (80)
Age 50–64 years	1286 (38)	1153 (90)	133 (10)
Age 65–79 years	1609 (47)	1395 (87)	214 (13)
Age ≥ 80 years	115 (3.4)	91 (79)	24 (21)
**T stage**			
cT1a	2674 (78)	2235 (87)	339 (13)
cT1b	735 (22)	671 (91)	64 (9.0)
Tumor size (cm) (mean, stdev)	3.2 (1.3)	3.2 (1.3)	2.9 (1.2)

**Table 2:** Multivariable logistic regression of factors predictive for benign histology.

Multivariable	Odds Ratio	95% Confidence Interval	*p*-Value
**Gender (male)**	0.925	0.744, 1.149	0.48
**Age category**			
<50 years (ref)	ref	-	-
50–64 years	1.391	0.928, 2.087	0.11
65–79 years	1.881	1.272, 2.781	0.002
≥80 years	3.642	2.030, 6.534	<0.001
**Tumor size**	0.793	0.724, 0.868	<0.001

**Table 3:** Summary of existing literature reporting benign pathology rate after partial nephrectomy for T1 tumors.

Author	Country	Study Period	Study Type	No. of Patients	Benign Pathology Rate
Kim et al., 2019 [17]	United States	2007–2014	National cohort	18,060	31%
Fernando et al., 2016 [3]	United Kingdom	2012	National cohort	1044	19%
Jeon et al., 2010 [12]	South Korea	1997–2008	Retrospective, single center	376	22%
Bauman et al., 2017 [16]	United States	2007–2015	Retrospective, single center	916	14%
Kim et al., 2020 [13]	Meta-analysis 47 studies	Studies up to 2019	Meta-analysis	3447	19%

## Data Availability

The data can be shared upon request.

## Supplementary Materials

The following supporting information can be downloaded at: https://www.mdpi.com/article/10.3390/cancers16203518/s1, Table S1: Histopathological subtypes of patients who underwent partial nephrectomy; Figure S1: Percentage of malignant versus benign pathologic findings after partial nephrectomy in The Netherlands.
