# Supplementary material for: A National Study of the Rate of Benign Pathology After Partial Nephrectomy for T1 Renal Cell Carcinoma: Should We Be Satisfied?
Source: Cancers (Basel). 2024 Oct 17;16(20):3518. doi: 10.3390/cancers16203518 (PMC11506599; doi:10.3390/cancers16203518)
Supplement: Supplementary file 1 [file cancers-16-03518-s001.zip › cancers-3213652-supplementary.pdf]

Supplementary Table S1: Histopathological subtypes of patients who underwent partial nephrectomy.

Subtype	Cohort (n=3409)
ccRCC	2126 (62)
pRCC	603 (18)
chRCC	178 (5.2)
Combination	42 (1.2)
Other RCC	57 (1.7)
Oncocytoma	345 (10)
AML	44 (1.3)
Other benign	14 (0.4)

Abbreviations: ccRCC: clear cell renal cell carcinoma, pRCC: papillary renal cell carcinoma, chRCC: chromophobe renal cell carcinoma, AML: angiomyolipoma. Other benign includes papillary adenoma, metanephric adenoma, and leiomyoma.

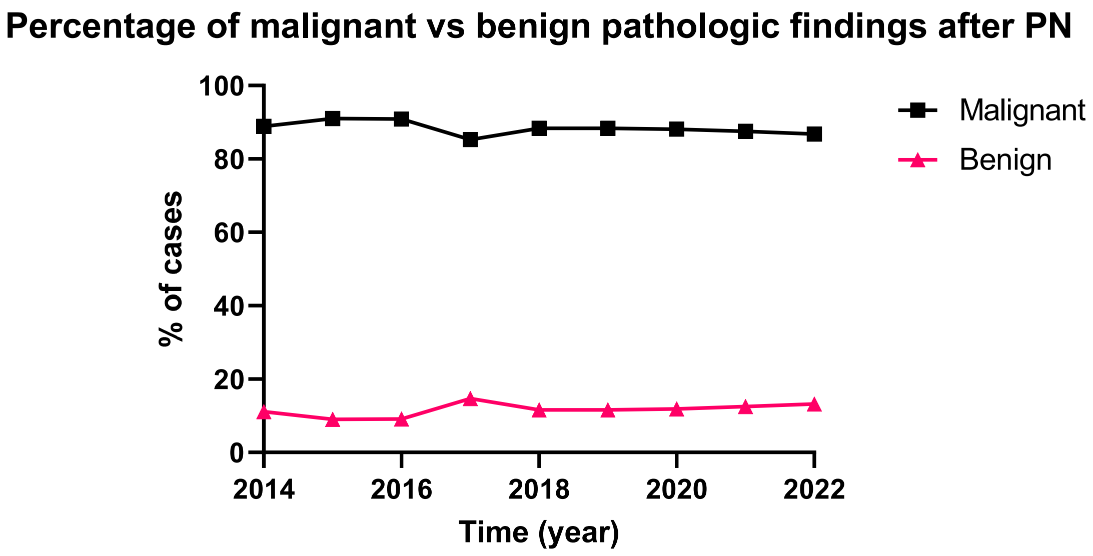

Supplementary Figure S1: Percentage of malignant versus benign pathologic findings after partial nephrectomy in The Netherlands.
